# Appetite Control across the Lifecourse: The Acute Impact of Breakfast Drink Quantity and Protein Content. The Full4Health Project

**DOI:** 10.3390/nu12123710

**Published:** 2020-11-30

**Authors:** Daniel R. Crabtree, William Buosi, Claire L. Fyfe, Graham W. Horgan, Yannis Manios, Odysseas Androutsos, Angeliki Giannopoulou, Graham Finlayson, Kristine Beaulieu, Claire L. Meek, Jens J. Holst, Klaske Van Norren, Julian G. Mercer, Alexandra M. Johnstone, on behalf of the Full4Health-Study Group

**Affiliations:** 1 Centre for Health Science, Division of Biomedical Sciences, University of the Highlands and Islands, Old Perth Road, Inverness IV2 3JH, UK; 2 The Rowett Institute, University of Aberdeen, Foresterhill Road, Aberdeen AB25 2ZD, UK; william.buosi.m@gmail.com (W.B.); c.fyfe@abdn.ac.uk (C.L.F.); j.mercer@abdn.ac.uk (J.G.M.); alex.johnstone@abdn.ac.uk (A.M.J.); 3 Biomathematics and Statistics Scotland, Foresterhill Road, Aberdeen AB25 2ZD, UK; g.horgan@abdn.ac.uk; 4 Department of Nutrition-Dietetics, School of Health Science & Education, Harokopio University Athens, 70 El. Venizelou Avenue, 17671 Kallithea, Greece; manios@hua.gr (Y.M.); agiann@hua.gr (A.G.); 5 Department of Nutrition and Dietetics, School of Physical Education, Sport Science and Dietetics, University of Thessaly, 42100 Trikala, Greece; oandroutsos@uth.gr; 6 School of Psychology, University of Leeds, Leeds LS2 9JT, UK; g.s.finlayson@leeds.ac.uk (G.F.); k.beaulieu@leeds.ac.uk (K.B.); 7 Institute of Metabolic Science, Metabolic Research Laboratories, University of Cambridge, Addenbrooke’s Hospital, Box 289, Hills Road, Cambridge CB2 0QQ, UK; clm70@cam.ac.uk; 8 Department of Biomedical Sciences and Novo Nordisk Foundation Center for Basic Metabolic Research, University of Copenhagen, DK-2200 Copenhagen, Denmark; jjholst@sund.ku.dk; 9 Nutritional Biology, Human Nutrition and Health, Wageningen University, 6708 WE Wageningen, The Netherlands; klaske.vannorren@wur.nl

**Keywords:** appetite, lifecourse, gut hormones, hunger, protein

## Abstract

Understanding the mechanisms of hunger, satiety and how nutrients affect appetite control is important for successful weight management across the lifecourse. The primary aim of this study was to describe acute appetite control across the lifecourse, comparing age groups (children, adolescents, adults, elderly), weight categories, genders and European sites (Scotland and Greece). Participants (*n* = 391) consumed four test drinks, varying in composition (15% (normal protein, NP) and 30% (high protein, HP) of energy from protein) and quantity (based on 100% basal metabolic rate (BMR) and 140% BMR), on four separate days in a double-blind randomized controlled study. Ad libitum energy intake (EI), subjective appetite and biomarkers of appetite and metabolism (adults and elderly only) were measured. The adults’ appetite was significantly greater than that of the elderly across all drink types (*p* < 0.004) and in response to drink quantities (*p* < 0.001). There were no significant differences in EI between age groups, weight categories, genders or sites. Concentrations of glucagon-like peptide 1 (GLP-1) and peptide YY (PYY) were significantly greater in the elderly than the adults (*p* < 0.001). Ghrelin and fasting leptin concentrations differed significantly between weight categories, genders and sites (*p* < 0.05), while GLP-1 and PYY concentrations differed significantly between genders only (*p* < 0.05). Compared to NP drinks, HP drinks significantly increased postprandial GLP-1 and PYY (*p* < 0.001). Advanced age was concomitant with reduced appetite and elevated anorectic hormone release, which may contribute to the development of malnutrition. In addition, appetite hormone concentrations differed between weight categories, genders and geographical locations.

## 1. Introduction

Nutrition-related noncommunicable diseases are associated with increased morbidity and mortality at all stages of life [1,2]. Physiological and psychological responses to food change as we age, with impact on food choices and preferences, but little is known about how appetite control varies across the lifecourse [3]. This is a critical issue in combatting food intake-related chronic disease, commonly driven by over-consumption, but also in consideration of relative under-nutrition in the elderly and the clinically compromised.

Food intake and appetite are governed across the lifecourse by complex interactions between peripherally synthesized gut hormones and their central receptors [4]. These interactions are subject to external influences, including hedonic cues and the environment [5]. Short-acting gastrointestinal signals include the anorexigenic peptides glucagon-like peptide 1 (GLP-1) and peptide YY (PYY) and the orexigenic hormone ghrelin, while leptin maintains long-term energy homeostasis [6]. Homeostatic systems can, however, be overridden by hedonic signals, resulting in appetite control dysfunction, excess energy consumption and obesity [7]. There may be key periods in the lifecourse when appetite can be modulated for optimal health. For example, the onset of overweight and obesity starts as early as childhood and can track into adulthood [8]. Environmental factors contribute towards weight gain, if rewarding energy-dense foods are freely available and integrated into local culture, creating an obesogenic environment [9]. With advancing age, food reward signals are altered [10,11], food craving behavior declines, particularly in females [12] and food intake is suppressed [13,14], all contributing to a condition termed the “anorexia of ageing” [15]. Cross-sectional research reports a peak in calorie intake during late adolescence, followed by a decline, with calorie intake reducing by 1300 kcal/day on average between 20 and 80 years of age for males and 600 kcal/day for females [16]. Understanding how dietary interventions influence physiological and behavioral mediators of appetite at different stages of life is vital for effective long-term weight control [17]. High-protein diets are often recommended for weight management, as they are highly satiating [18,19,20], and in the prevention and treatment of malnutrition, particularly in elderly populations [21]. Protein-induced satiety has been observed acutely, within single meals that contained 25% to 81% of energy from protein, associated with reductions in subsequent energy intake (EI) compared to lower protein alternatives [22]. In children and adolescents, studies have reported either an appetite suppressant effect of increased protein content [23] or no effect [24]. In addition, breakfasts high in protein have been shown to induce greater hunger suppression compared to breakfasts with a lower protein content in adults [25]. It is not well understood how interactions between protein and appetite control differ between children, adolescents, adults and the elderly since studies are rarely conducted across the lifecourse.

The primary aim of this study was to describe the acute regulation of appetite across the lifecourse, thus being able to detect differences between four different age groups (children, adolescents, adults and elderly), two different weight categories (normal weight and overweight), the two genders (male and female) and two European sites (Aberdeen, Scotland and Athens, Greece). The secondary aim was to examine the short-term effects of breakfast test drinks varying in protein composition and quantity on appetite control. Our study is unique in that it applies an individualized appetite challenge across the lifecourse in lean and overweight males and females in northern and southern Europe.

## 2. Materials and Methods

### 2.1. Participants

Normal weight and overweight/obese and male and female child, adolescent, adult and elderly participants (age range 7–77 years) were recruited in Scotland and Greece as part of an identical, dual-site within-day dietary intervention study, thereby creating four groups: age (children, adolescents, adults and elderly), weight category (normal weight and overweight), gender (male and female) and site (Scotland and Greece). Recruitment of volunteers was by public advertisement using radio, newspapers and social media, and was conducted from May 2012 to August 2015. When requested, study information sessions were conducted at schools, health care centers and day care centers for the elderly. Participants were individuals who were motivated to actively respond to the volunteer request. Exclusion criteria included: smokers; morbid obesity (BMI ≥ 40 kg/m^2^); pregnancy; obesity of known endocrine origin; neurological disorders; medication known to influence appetite (including orlistat, oral antidiabetics, insulin, digoxin, anti-arrhythmics, sibutramine, antidepressants); self-reported fever/systemic infection; participation in medical or surgical weight loss program within 1 month of selection; history of cerebrovascular disease; current major depressive disorder; history of cardiovascular disease; chronic obstructive pulmonary disease; an allergy to any of the test drink components and partaking in >6 h of vigorous physical activity per week. This study was conducted according to the guidelines laid down in the Declaration of Helsinki [26]. Ethical approval in Aberdeen was granted by the National Health Service North of Scotland Research Ethics Service. Ethical approval in Athens was granted by the Bioethics Committee of Harokopio University and the Greek Ministry of Education for the implementation of the study in schools. The study received ethical approval from NHS Grampian, Aberdeen, Scotland, UK and the Research Ethics Committee (reference number: 12/NS/0007). All participants provided written informed consent before entering the study and, in addition, the parents/guardians of the children consented for their child to participate.

### 2.2. Experimental Procedures and Protocol

**Figure 1:**
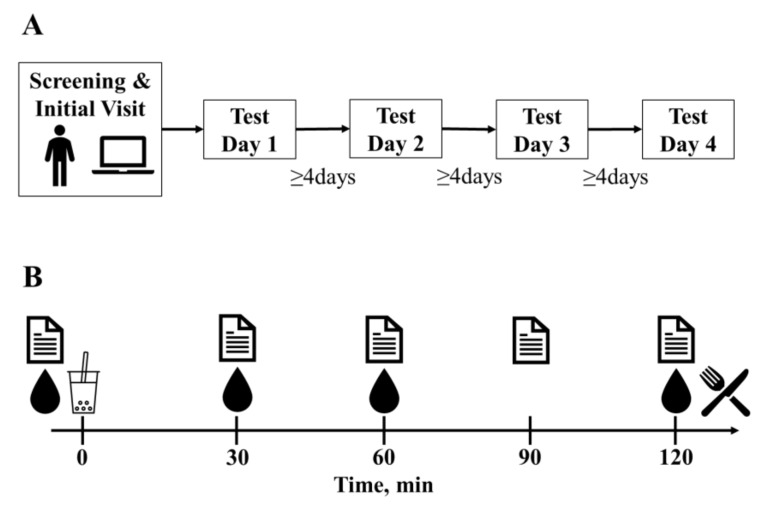
Experimental design (**A**) and test day protocol (**B**). 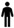 anthropometric measurements, 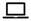 LFPQ, 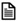 appetite ratings (Likert scale, children and adolescents; VAS, adult and elderly), 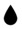 blood sampling (adult and elderly cohorts only), 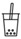 randomized test drink intake (NPMT, NPWL, HPMT or HPWL), 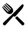 ad libitum food intake from buffet-style test meal; (HPMT) high-protein maintenance, HPWL: high-protein weight loss, LFPQ: Leeds Food Preference Questionnaire, NPMT: normal-protein maintenance, NPWL: normal-protein weight loss, VAS: visual analog scale.

Data on children and adolescents were collected at schools and adults and elderly attended the Rowett Institute, University of Aberdeen, Scotland (ABDN) and the Department of Nutrition-Dietetics, Harokopio University Athens, Greece (HUA). Prior to the main experimental trials, preliminary anthropometric measures were carried out under standardized conditions. During the main experimental trials, participants consumed four test drinks for breakfast on four separate occasions using a double-blind randomized controlled crossover design, with at least a 4 day period between trials. On the morning of each trial, participants arrived following an overnight fast (10 h) and having refrained from alcohol consumption and strenuous exercise for 12 h. Test drinks were consumed immediately following baseline measures (0 min), then at 120 min post-baseline, ad libitum EI was measured by means of a 30 min buffet-style test meal, after which participants were free to leave. Subjective appetite sensations were assessed using visual analog scales (VASs) at 0, 30, 60, 90 and 120 min (pre-meal), pleasantness and satisfaction VASs were also completed immediately post-test drink. Blood samples were taken at 0, 30, 60 and 120 min to determine biomarker concentrations. The true aims of the study were concealed from the participants; however, all participants were fully debriefed following their completion of the study. See Figure 1 for an overview of the experimental protocol. This trial was registered at clinicaltrials.gov as NCT01597024.

### 2.3. Anthropometric Measures

Height, body mass, waist circumference and body composition were measured in the fasted state and after voiding as described previously [27]. Height was measured to the nearest 0.1 cm using a portable stadiometer (Model 213, SECA, Hamburg, Germany). Body mass, measured to the nearest 0.1 kg, and body composition were assessed using a multi frequency segmental body composition analyzer (Model BC-418-MA, Tanita Corporation, Tokyo, Japan). Body mass index was calculated for each participant and compared against the age- and gender-matched thresholds for normal weight and overweight, as defined by the World Health Organization [28]. In addition, waist circumference and visceral fat percentage measures were performed using abdominal bioelectrical impedance analysis (AB 140 Viscan, Tanita Corporation, Tokyo, Japan), with participants in the supine position.

### 2.4. Test Drinks

The test drinks provided for the study were designed by Nutricia (Danone, Utrecht, The Netherlands) to taste, look and smell identical. One test drink was created with a normal-protein (NP) composition (15% energy from protein) and the other was created with a high-protein (HP) composition (30% energy from protein). The test drink compositions are presented in Table 1 and compared with whole milk. The test drink quantity either corresponded to 100% of the participant’s estimated basal metabolic rate (BMR, kcal/day; weight loss requirements, WL) or 140% of the participant’s estimated BMR (weight maintenance requirements, MT). Basal metabolic rate was estimated for all age groups according to equations derived by Schofield [29], suitable for children and adults (see Table S1 for the equations used to estimate BMR). When calculating participant energy requirements for the MT drinks, BMR was multiplied by a correction factor of 1.4, whereas when calculating energy requirements for the WL drinks, BMR was multiplied by a correction factor of 1. In addition, for the purposes of this study, breakfast was defined as the first meal of the day consisting of 25% of the participant’s daily energy requirements, which is similar to previous studies [30,31]. Therefore, when calculating participant energy requirements for both drinks, daily BMR was multiplied by 0.25. The following formulas give the energy requirements (ER) for the MT and WL test drinks, respectively:

ER_MT_ = BMR × 1.4 × 0.25 (1)

ER_WL_ = BMR × 1 × 0.25 (2)

The quantity of each drink to be served was calculated considering the energy density of the drinks. The energy density of the drinks in kcal/100 mL (ED_kcal/100 mL_) was:

ED_kcal/100 mL_ = 130 kcal/100 mL (3)

The physical density (d) of the drinks was 1.088 kg/L. Therefore, the energy density of the drinks in kcal/100 g (ED_kcal/100 g_) was:

ED_kcal/100 g_ = (ED_kcal/100 mL_ ÷ d × 100) × 100 (4)

ED_kcal/100 g_ = 130 ÷ 1.09 = 119 kcal/100 g (5)

Finally, the quantity of drink to be served was as follows:

Quantity (g) = (ER ÷ ED_kcal/100 g_) × 100 = (ER ÷ 119) × 100 (6)

Each participant consumed the four different types of test drink: normal-protein weight loss (NPWL), normal-protein weight maintenance (NPMT), high-protein weight loss (HPWL) and high-protein weight maintenance (HPMT) in a randomized order and at a standardized time. The composition of the test drinks was double-blinded and the drinks were labeled A, B, C and D. Nutricia labeled the drinks and generated the random allocation sequence. The drinks were weighed to the nearest gram and placed into neutral sealed cups with a straw. Participants were required to consume at least 80% of each drink and failure to do so would result in their withdrawal from the study. The composition of the drinks was unblinded to the researchers after the final participant completed the study.

### 2.5. Ad Libitum EI

The ad libitum buffet-style test meal consisted of a counter-balanced selection of 25 sweet and savory, high- and low-calorie food and drink items, all of which were provided in excess (Table S2). All food and drink items were chosen to be commercially available in the UK and Greece. Buffet items were offered either in transparent plastic containers or in their original packaging. The buffet was provided 120 min after test drink consumption, participants were given access to the buffet for 30 min and instructed to consume as much or as little of each buffet item as they wanted until they were satisfied. All foods and drinks were presented identically on each occasion and covertly weighed before and after the buffet. Ad libitum energy and macronutrient intakes were calculated using nutritional values provided by the manufacturer, or by using an electronic version of McCance and Widdowson’s The Composition of Foods [32]; NETWISP™ software (version 3.0 for Windows, Tinuviel Software, Anglesey, UK).

### 2.6. Subjective Appetite Assessment

Appetite perceptions (hunger, fullness and prospective food consumption (PFC)) were measured in adult and elderly participants using previously validated 100mm visual analog scales (VASs, [33]). Participants indicated their subjective feelings of appetite by marking a vertical line on the VAS. A composite appetite score was calculated at each time of measurement using the following formula:

[Hunger + (100 − fullness) + prospective consumption]/3(7)

Higher composite appetite scores relate to elevated feelings of appetite. The composite appetite score is increasingly used in the literature for ease of data analysis and presentation [25,34].

Children and adolescents used a 9-point Likert scale to rate fullness (How full do you feel?) and PFC (How much do you think you could eat now?), with 1 representing “Not at all full”/”Nothing at all” and 9 representing “As full as I’ve ever felt”/”A large amount” for fullness and PFC, respectively. Participants were not permitted to view their previous ratings when completing the scales.

### 2.7. Test Drink Pleasantness and Satisfaction

After consuming the test drinks, participants rated the drinks for pleasantness and satisfaction. The adult and elderly participants used a 100 mm VAS to rate the drinks, with “Not at all pleasant”/”Not at all satisfying” on the left side and “Extremely pleasant”/”Very satisfying” on the right side of the pleasantness and satisfaction scales. Children and adolescents used a 9-point Likert scale adapted from Jansen et al. [35] to rate the drinks for pleasantness and satisfaction. The scale consisted of 5 cartoon faces (smileys). The first cartoon face on the left (unhappy) reflecting low perceived pleasantness/satisfaction was scored 1 and the last face (very happy) on the right of the scale, reflecting high perceived pleasantness/satisfaction, was scored 9. Participants could rate in between two faces, creating a 9-point scale.

### 2.8. Food Reward: Leeds Food Preference Questionnaire (LFPQ)

The Leeds Food Preference Questionnaire [36] provided a baseline measure of liking and wanting along dimensions of fat and taste. Participants were presented with an array of pictures of individual food items common in the diet. Foods were chosen by the local research team from a validated database to be either predominantly high (>50% energy) or low (<20% energy) in fat, sweet or savory in taste, but similar in familiarity, protein content and cultural suitability for the study population [37]. The LFPQ has been validated in previous studies investigating dietary protein [38,39,40]. Explicit liking was measured by participants rating the extent to which they liked each food using a 100 mm VAS (“How pleasant would it be to taste this food now?”). Implicit wanting was assessed using a forced choice methodology so that every image from each of the four food types was compared to every other type over 96 trials (food pairs). Reaction times for all responses were covertly recorded for each food type after adjusting for frequency of selection [37]. Fat bias scores for liking and wanting were calculated as the difference between the high-fat scores and the low-fat scores. Sweet bias scores were calculated as the difference between the sweet and savory scores. Positive values indicated greater liking/wanting for high fat > low fat or sweet > savory and negative values indicated the reverse. 

### 2.9. Blood Sampling and Processing

At all test visits, glucose, insulin, total ghrelin, PYY and GLP-1 were measured fasted and postprandially (at 30, 60 and 120 min after eating), while leptin was measured at the first test visit only. An intravenous cannula (BD Venflon, BD, UK) was inserted into an antecubital vein for the collection of venous blood samples. During the trials, the cannula was kept patent with 2mL flushes of 0.9% NaCl(aq) isotonic saline solution (Baxter Healthcare, UK) after each bloodletting. At each time point, a venous blood sample was collected into a 4.9mL EDTA-coated monovette (S-Monovette, Sarstedt, Nümbrecht, Germany) for the measurement of plasma total ghrelin, PYY and GLP-1 concentrations. A second venous blood sample was collected into a 2.7 mL lithium heparin-coated monovette (S-Monovette, Sarstedt, Nümbrecht, Germany) for the measurement in plasma of leptin, glucose and insulin. Immediately after blood collection, collection tubes were placed in ice and 160µL of a preservative containing 4-(2-aminoethyl) benzenesulfonylfluoride hydrochloride (Roche, Basel, Switzerland), dipeptidyl peptidase-4 inhibitor (Merck Millipore, Darmstadt, Germany) and protease inhibitor cocktail (Sigma-Aldrich, St. Louis, MO, USA) were added to EDTA-coated monovettes. After gentle inversion, both monovettes were spun at 1000g for 15 min in a centrifuge at 4 °C and plasma was stored at −80 °C for batch analysis at the conclusion of the study. Identical blood sampling and processing procedures were followed at ABDN and HUA. Blood samples were collected from adult and elderly participants only. No samples were collected from children, as gaining ethical approval for blood samples in this vulnerable group was challenging.

### 2.10. Biomarker Analysis

#### 2.10.1. Appetite Hormones

Total ghrelin concentrations were measured using a human-specific radioimmunoassay kit (Linco Research, St. Charles, MO, USA) at the laboratory of JJ Holst. The lowest concentration of ghrelin detectable using this assay was 93 pg/mL. The limit of linearity for this assay was 6000 pg/mL. All samples were read using a gamma counter. The between- and within-volunteer CVs were 39% and 14%, respectively. Total PYY and GLP-1 were measured in duplicate using an electrochemical luminescence immunoassay kit (Meso Scale Discovery, Rockville, Maryland, USA) on the Meso Scale Discovery^®^ multiarray assay platform (Meso Scale Discovery, Rockville, Maryland, USA) at the Core Biomedical Assay Laboratory (CBAL), Cambridge. The PYY immunoassay measured both PYY_1–36_ and PYY_3–36_ with a range of 30–3000 pg/mL. Inter-assay CVs of 7.8–16.4% were obtained. The GLP-1 immunoassay measures all endogenous forms of GLP-1 (including GLP-11-36, GLP-11-37, GLP-17-36, GLP-17-37, GLP-19-36 and GLP-19-37) and has a range of 1.4–1000 pg/mL and CVs of 5.2–8.2% for most of the analytical range. Leptin analysis was performed at CBAL using an in-house two-site DELFIA^®^ assay, which used a monoclonal capture antibody and a polyclonal detection antibody with fluorescent detection using europium-labeled streptavidin [41,42]. The antibodies and standards were sourced from R&D Systems (R&D Systems Europe, Abingdon, UK). This assay had a lower limit of detection of 0.1 ng/mL and intra-assay CVs of 3.9–7.1%.

#### 2.10.2. Glucose Homeostasis

Glucose and insulin plasma analysis was conducted at the University of Aberdeen, Rowett Institute, Technical Services department. Glucose concentrations were measured using a hexokinase method on a Dimension^®^ clinical chemistry analyzer (Siemens Healthcare GmbH, Erlangen, Germany) with CVs of <2% within the reference range. Insulin was detected using a Liaison^®^ XL automated immunoassay analyzer (DiaSorin, Italy) with a chemiluminescence immunoassay, which had a range of 20–3470 pmol/L and intra-assay CVs of 5.0–6.0% across the analytical range. The homeostatic model assessment [43] was used to estimate hepatic insulin resistance (HOMA-IR):

IR_insulin_: fasting glucose (mmol/L) × fasting insulin (mU/L)/22.5 (8)

β-cell function was measured using an early insulin secretion function index (insulinogenic index (IGI)):

IGI: (Insulin_0 min − Insulin_30 min)/(Glucose_0 min − Glucose_30 min)(9)

Insulin to glucose ratio (IGR) was also calculated.

### 2.11. Statistical Analysis

It was calculated that 16 participants in each group (defined by age, BMI, gender and study site) would give approximately 80% power to detect group and treatment differences in any variable comparable to the unpredictable variation between groups or within individuals, i.e., to detect a standard effect size of approximately 1.0. Main factor effect comparisons are based on combinations of groups and so larger volunteer numbers had the power to detect smaller effect sizes.

Variables were analyzed by linear mixed models using residual maximum likelihood with random effect terms for volunteer and fixed effect terms for test drink type, age group, BMI group, gender, site and all two-way and three-way interactions. An additional analysis was carried out in each case in which the drink effect was decomposed into its factorial components of composition (HP vs. NP) and quantity (WL vs. MT). Where data were collected at several timepoints in a day (appetite scores and gut hormones), an additional random effect term for day, and fixed effect term for time, were included in the models. Significance of fixed effect terms was assessed by F statistics calculated from Wald statistics, with estimated denominator degrees of freedom. Drinks were compared with post hoc tests based on least significant differences. A *p* value < 0.05 was considered to indicate statistical significance. Analyses were carried out using Genstat v17 (VSN International, Hemel Hempstead, UK). Data are presented as mean ± the standard error of the differences of the mean (SED) unless stated otherwise.

## 3. Results

### 3.1. Participants

In total, 424 members of the public were enrolled in the study (See the Consolidated Standards of Reporting Trials (CONSORT) flow diagram (Figure S1) summarizing the participant flow). Thirty-three participants discontinued the study after randomization, of which five were excluded as they consumed <80% of at least one of the test drinks. Therefore, 391 participants across ABDN and HUA completed the study, as 103 children, 109 adolescents, 97 adults and 82 elderly. The characteristics of the participants from ABDN and HUA who completed the study are presented in Table 2. In addition, Supplementary Materials Table S3 presents the number of participants allocated to each group at both sites.

### 3.2. Test Drinks

The average test drink energy (kcal) and protein (g) consumption, corrected for mass consumed, varied significantly between age groups (*p* < 0.001; Table 3).

### 3.3. Ad Libitum EI

Differences in mean ad libitum EI after the test drinks between age groups, weight categories, genders and sites are presented in Table 4. Differences between age groups in response to the quantity of drink provided (WL vs. MT) approached significance (*p* = 0.074).

There were no significant differences between weight categories, genders or sites. Furthermore, there were no significant differences in total caloric intake (test drink EI + ad libitum EI) between age groups, weight categories, genders or sites in response to drink type, composition or quantity (Table 5).

The data for mean ad libitum energy and macronutrient intake with all participants combined are reported in Table S4, to explore drink effects. Ad libitum EI was significantly greater after consuming the NPWL drink, in comparison to the other drink types (*p* < 0.001). There were small but statistically significant differences in energy and macronutrient intakes between drinks fed at WL or MT quantities, reflected by higher intakes after WL (all *p* < 0.001). There were no differences in ad libitum energy or nutrient intakes between the NP and HP drinks. 

Visit number had a significant effect on ad libitum EI, with EI significantly greater for visits 2, 3 and 4 compared to visit 1 for all participants combined (Supplementary Materials Table S5*; p* = 0.001). In addition, the effect of visit number on ad libitum EI differed significantly between age groups (*p* < 0.001).

### 3.4. Subjective Appetite Assessment

Table 6 presents the fullness and PFC ratings for drink type, composition and quantity x time interactions for children and adolescents. Fullness did not differ between children and adolescents in response to drink type (*p* = 0.252), composition (*p* = 0.220) or quantity (*p* = 0.554). There were no significant differences in ratings of PFC in response to drink type (*p* = 0.332), composition (*p* = 0.209) or quantity (*p* = 0.653) when comparing children and adolescents. There were no significant differences in fullness or PFC ratings between weight categories, genders or sites (data not shown).

Table 7 presents the composite appetite score for drink type, composition and quantity x time interactions for adults and elderly. The adults’ appetite score was significantly greater than that of the elderly across all drink types (*p* < 0.004) and in response to both drink quantities (*p* < 0.001). There were no significant differences between adult and elderly appetite scores in response to drink composition (*p* = 0.624). There were no significant differences in composite appetite scores between weight categories, genders or sites (data not shown).

### 3.5. Test Drink Pleasantness and Satisfaction

There was a significant difference in pleasantness ratings between drinks for the children and adolescents and the adults and elderly, with a significantly higher rating for the NPWL drink, in comparison to the NPMT, HPWL and HPMT drinks (children and adolescents (*n* = 212): 5.14, 4.80, 4.97, 4.60, respectively; *p* < 0.001; SED: 0.12; adults and elderly (*n* = 179): 68.4, 64.6, 64.7, 62.5 mm, respectively; *p* = 0.002; SED: 1.9). Participants also preferred the NP over HP composition (children and adolescents: 4.97, 4.78, respectively; *p* = 0.032; SED: 0.09; adults and elderly: 66.5, 63.6 mm, respectively; *p* = 0.012; SED: 1.3) and the WL over the MT quantity (children and adolescents: 5.05, 4.70, respectively; *p* < 0.001; SED: 0.09; adults and elderly: 66.5, 63.6 mm, respectively; *p* = 0.04; SED: 1.3). Average pleasantness ratings were significantly higher in the ABDN children and adolescent cohort (*n* = 84) compared to the HUA cohort (*n* = 128; 5.86, 3.89, respectively; *p* < 0.001; SED: 0.22).

Average satisfaction ratings were significantly higher in the children (*n* = 103) compared to the adolescents (*n* = 109; 5.17, 4.27, respectively; *p* = 0.009; SED: 0.20). Children and adolescent females (*n* = 107) reported significantly higher average satisfaction ratings compared to males (*n* = 105; 4.95, 4.49, respectively; *p* = 0.037; SED: 0.20), and average satisfaction ratings were higher in the ABDN children and adolescent cohort (*n* = 84) compared to the HUA cohort (*n* = 128; 5.45, 3.99, respectively; *p* < 0.001; SED: 0.20). There were no differences between age groups, weight categories, genders or sites and no effect of drink on satisfaction ratings in the adults and elderly (*n* = 179; data not shown).

### 3.6. Food Reward: LFPQ

Fat bias scores (liking and wanting scores for high-fat relative to low-fat foods) and sweet bias scores (scores for sweet relative to savory foods) were compared according to age, BMI, gender and site (Table S6). There was a main effect of age group on liking (*p* = 0.001) and wanting (*p* < 0.001) for high-fat food. Post hoc analyses showed that the elderly had the lowest fat preference, followed by adults, and that both groups showed a clear preference (liking and wanting) for low-fat relative to high-fat foods. Adolescents showed a greater liking and wanting for high-fat relative to low-fat food. There was also an effect of age group on wanting for sweet foods (*p* < 0.001), with a greater wanting for sweet in children, adolescents and elderly compared to adults (*p* < 0.05). For BMI, while there were no group differences for liking and wanting fat bias, liking (*p* = 0.047) and wanting (*p* = 0.059) sweet bias tended to be greater in normal weight than overweight participants. There was no main effect of gender. There was a main effect of site on liking and wanting for sweet (both *p* = 0.019) and high-fat (both *p* < 0.001) foods, with the ABDN population showing a greater sweet and fat preference compared to HUA. 

### 3.7. Biomarkers

Note that, of the 179 adult and elderly participants, four normal weight adults and three normal weight elderly from ABDN were unable to provide blood samples, therefore, 172 participants were included in the biomarker analyses.

#### 3.7.1. Appetite Hormones

**Figure 2:**
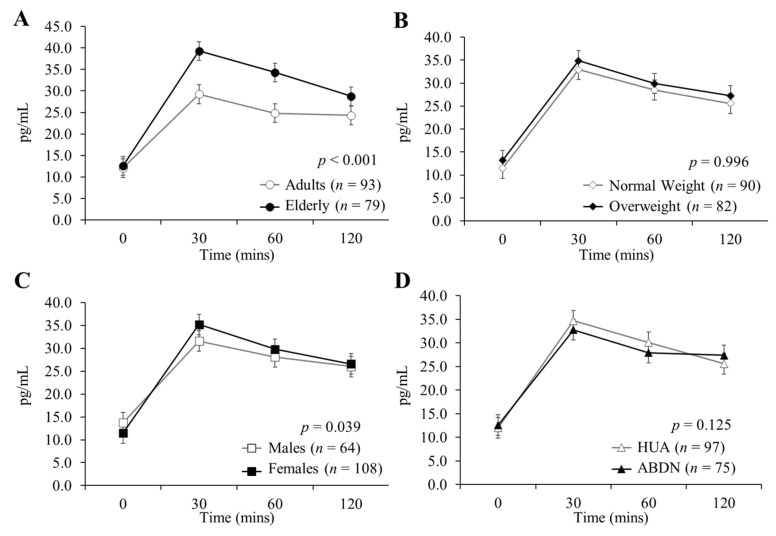
Plasma concentrations of GLP-1 in the adult and elderly cohorts in response to all test drinks combined. Data are presented as mean ± SED, *n* = 172; seven ABDN participants (four normal weight adults, three normal weight elderly) did not complete this measurement. Determined using an electrochemical luminescence immunoassay kit, values were analyzed as repeated measurements using ANOVA, differences are statistically significant when *p* < 0.05. (**A**) Comparison of age group, (**B**) comparison of BMI group, (**C**) comparison of gender, (**D**) comparison of site. ABDN: Scotland, GLP-1: glucagon-like peptide 1, HUA: Greece.

**Figure 3:**
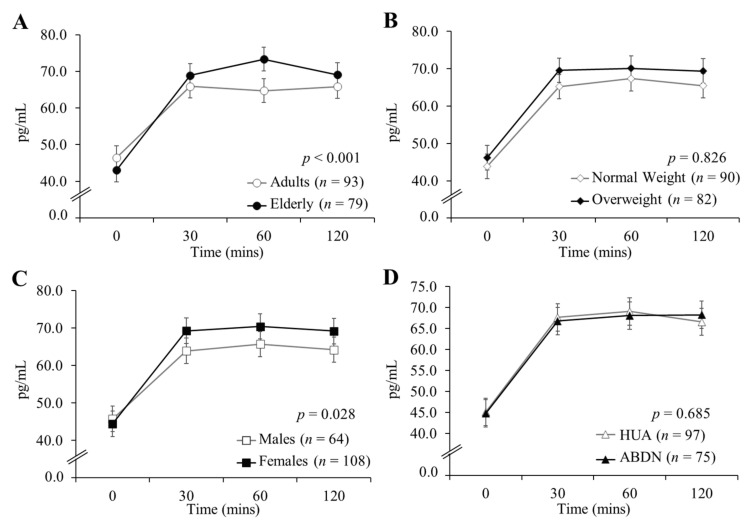
Plasma concentrations of PYY in the adult and elderly cohorts in response to all test drinks combined. Data are presented as mean ± SED, *n* = 172; seven ABDN participants (four normal weight adults, three normal weight elderly) did not complete this measurement. Determined using an electrochemical luminescence immunoassay kit, values were analyzed as repeated measurements using ANOVA, differences are statistically significant when *p* < 0.05. (**A**) Comparison of age group, (**B**) comparison of BMI group, (**C**) comparison of gender, (**D**) comparison of site. ABDN: Scotland, HUA: Greece, PYY: peptide YY.

**Figure 4:**
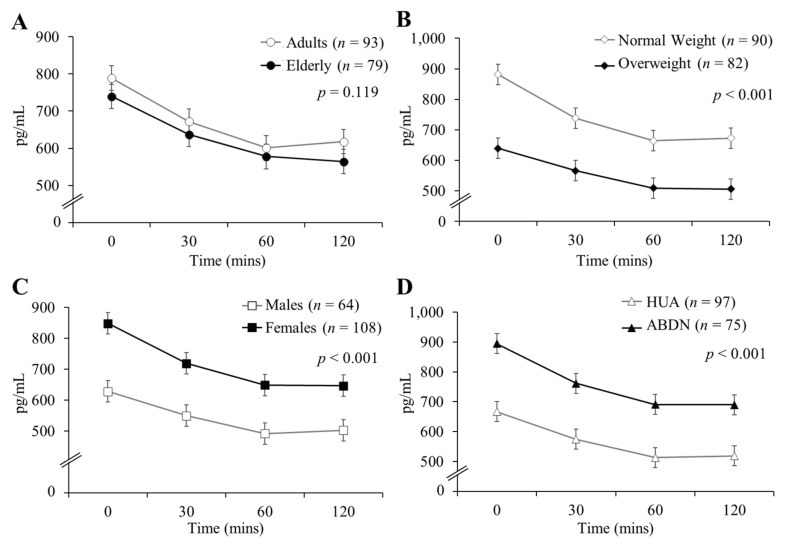
Plasma concentrations of ghrelin in the adult and elderly cohorts in response to all test drinks combined. Data are presented as mean ± SED, *n* = 172; seven ABDN participants (four normal weight adults, three normal weight elderly) did not complete this measurement. Determined using a human-specific radioimmunoassay kit, values were analyzed as repeated measurements using ANOVA, differences are statistically significant when *p* < 0.05. (**A**) Comparison of age group, (**B**) comparison of BMI group, (**C**) comparison of gender, (**D**) comparison of site. ABDN: Scotland, HUA: Greece.

Differences in GLP-1, PYY and ghrelin concentrations between age groups, weight categories, genders and sites in response to all test drinks combined are presented in Figure 2 (GLP-1), Figure 3 (PYY) and Figure 4 (ghrelin). GLP-1 and PYY baseline concentrations did not differ significantly between groups for age, BMI, gender or site comparisons. Ghrelin baseline concentrations did not differ significantly between age groups, but baseline differences are reported for gender, BMI and site comparisons (all *p* < 0.001). Plasma concentrations of GLP-1 (Figure 2A) and PYY (Figure 3A) were significantly greater in the elderly than the adults (both *p* < 0.001), however, there were no significant differences in ghrelin release between age groups (Figure 4A; *p* = 0.119). There were no significant differences in GLP-1 (Figure 2B; *p* = 0.996) or PYY (Figure 3B; *p* = 0.826) responses between normal weight and overweight participants, however, normal weight participants exhibited significantly greater ghrelin concentrations when compared to overweight participants (Figure 4B; *p* < 0.001). Concentrations of all three hormones were greater in females in comparison to males (*p* = 0.039, *p* = 0.028 and *p* < 0.001 for GLP-1 (Figure 2C), PYY (Figure 3C) and ghrelin (Figure 4C), respectively). There were no differences in GLP-1 (Figure 2D) or PYY (Figure 3D) concentrations between ABDN and HUA participants, though ghrelin concentrations were greater in the ABDN cohort compared to the HUA cohort (Figure 4D; *p* < 0.001). Interestingly, ghrelin differences between sites could not be explained by differences in body composition or gender. 

Table 8 presents the gut hormones (GLP-1, PYY and ghrelin) drink type, composition and quantity x time interactions for all participants combined. There was a significant effect of drink type on concentrations of GLP-1 and PYY (both *p* < 0.001) and ghrelin (*p* < 0.005). The HP test drinks elicited a significantly greater increase in GLP-1 and PYY (both *p* < 0.001) in comparison to the NP drinks, however, protein content did not significantly affect ghrelin (*p* = 0.710). The MT test drinks elicited a significantly greater increase in GLP-1 and PYY (both *p* < 0.001) in comparison to the WL drinks (both *p* < 0.001), while ghrelin was suppressed to a significantly greater extent in response to the MT drinks compared to the WL drinks (*p* < 0.001).

Pooled data from adults and elderly demonstrated that GLP-1 and PYY concentrations were negatively associated with ad libitum EI (both *p* < 0.001), while there was no significant association between ghrelin and ad libitum EI (*p* = 0.770). PYY concentrations were negatively associated with composite appetite score (*p* = 0.028), while the association between GLP-1 concentrations and composite appetite score approached significance (*p* = 0.052). There was no significant association between ghrelin concentrations and composite appetite score (*p* = 0.605).

There were no significant differences in fasting leptin concentrations between adult and elderly participants (adults: 18.21 ng/mL; elderly: 20.86 ng/mL; *p* = 0.408; SED: 2.08). It is well established that obesity enhances the synthesis and release of leptin and, as anticipated, leptin concentrations were significantly higher in overweight participants compared to normal weight participants (overweight: 29.49 ng/mL; normal weight: 10.26 ng/mL; *p* < 0.001; SED: 2.04). Females exhibited significantly greater concentrations of leptin than males (females: 26.55 ng/mL; males: 7.40 ng/mL; *p* < 0.001; SED: 2.12). Leptin concentrations were also significantly greater in HUA participants compared to the ABDN cohort (HUA: 21.36 ng/mL; ABDN: 16.93 ng/mL; *p* = 0.016; SED: 1.98). The gender and site differences can be explained by differences in body composition.

#### 3.7.2. Glucose Homeostasis

Table S7 presents the group x time interactions for glucose and insulin concentrations, and includes HOMA-IR, IGI and IGR. Elderly participants exhibited significantly greater concentrations of glucose (*p* < 0.001), insulin (*p* < 0.001) and HOMA-IR (*p* < 0.001) compared to adults. As expected, glucose homeostasis was significantly influenced by BMI, with glucose (*p* < 0.001), insulin (*p* < 0.001), HOMA-IR (*p* < 0.001) and IGR (*p* = 0.006) greater in overweight compared to normal weight participants. Glucose (*p* = 0.036) and IGR (*p* = 0.005) were significantly greater in females compared to males. Glucose (*p* = 0.008), insulin (*p* = 0.005), HOMA-IR (*p* = 0.017) and IGR (*p* = 0.005) were significantly greater in the HUA cohort compared to the ABDN cohort. These significant group × time interactions were due to delayed insulin responses in elderly, overweight and HUA participants. Furthermore, differences in insulin concentrations between sites can be explained by differences in body composition.

There were significant drink type (*p* < 0.001), composition (*p* < 0.05) and quantity (*p* < 0.001) × time interaction effects for glucose, insulin, HOMA-IR, IGI and IGR (Table S8).

## 4. Discussion

In relation to the primary aim of this study, the current novel findings demonstrate that ad libitum EI did not differ significantly between age groups, BMIs, genders or geographical locations, though composite appetite score was lower in the elderly subjects compared to the younger adults in response to drink type and quantity. The elderly group exhibited greater postprandial levels of GLP-1 and PYY than the adult group, but ghrelin release was not affected by age. Concentrations of all appetite hormones were greater in females compared to males, while ghrelin levels were lower and fasting leptin levels were higher in overweight compared to normal weight participants. Furthermore, elevated ghrelin release and suppressed fasting leptin levels were observed in the ABDN cohort in comparison to the HUA cohort. As regards the secondary aim, ad libitum EI was not affected by drink composition, though concentrations of GLP-1 and PYY were higher in response to the HP compared to the NP test drinks. In addition, as might be expected, in response to the WL drink quantity, ad libitum EI was elevated, GLP-1 and PYY levels were lower and ghrelin concentrations were higher in comparison to the MT drink quantity.

### 4.1. Ad Libitum EI and Subjective Appetite

In the present study, there were no significant effects of the test meal on ad libitum EI between weight categories, gender, site or age; albeit we noted a trend towards differences between age groups in response to the quantity of drink provided below or at maintenance requirements. This approached significance, in part explained by the lower intakes in the elderly participants, which was also detected in their significantly lower subjective appetite score. Other authors have highlighted differences in appetite suppression between young and older healthy participants in response to protein and energy load [44], and this warrants further investigation to explore the influence of ageing on mechanisms of protein-induced satiety. Although we assessed subsequent ad libitum EI 2 h after the breakfast drink, we did not measure 24 h EI, so it may be that energy compensation occurred later in the day. Belza et al. [25] also examined the effects of consuming an NP vs. HP test drink in adults. The HP drink led to reduced hunger and increased satiety compared to the NP drink. This might be because the HP drink that Belza et al. [25] used provided 50% energy from protein or 88.4g protein per dose; there may be a threshold absolute concentration of protein required to stimulate protein-induced satiety, and the amount of protein supplied in our HP drink (10 g) may have fallen below this threshold. Furthermore, food form could be important for appetite control across the lifecourse. Indeed, Leidy et al. [23] demonstrated that a solid meal reduced lunch intake by approximately 480 kJ compared to a liquid meal in adolescents. By the nature of our current study design, we did not investigate the form of protein delivery and it is unclear as to whether a solid version would elicit greater changes.

To the authors’ knowledge, this is one of the few studies to report the effects of study visit number on ad libitum EI. Interestingly, we observed that the children’s ad libitum EI was lower on the final visit compared to the first visit, in agreement with previous research [45], while the adult and elderly ad libitum EI was higher. Possible explanations for these findings may include children habituating to the buffet items following their initial, novel exposure to the buffet during visit 1, and the older age groups initially experiencing heightened feelings of anxiety before acclimatizing to the environment, as demonstrated previously [46]. Future studies may consider incorporating a familiarization/acclimatization session when assessing ad libitum EI, to reduce the effects of study visit order.

### 4.2. Food Reward: LFPQ

A greater liking and wanting for low-fat relative to high-fat foods and non-sweet relative to sweet foods was shown in the Greek participants. This difference may reflect in part the cultural norms for consuming sweet foods in the morning in Scotland but may also be due to the greater availability of fresh fruit and vegetables and the more traditional rather than “‘westernized” diet in Greece [47,48]. Indeed, a north–south European differentiation in food habits consistent with these findings has previously been proposed [49]. We also found an age effect, with adults and the elderly having a greater liking and wanting for low-fat food compared to children and adolescents, and adults having a lower wanting of sweet foods compared to similarly high sweet wanting scores in children, adolescents and the elderly. Very few studies have examined food preferences across the lifecourse and, to our knowledge, no studies have examined both dimensions of fat and sweet taste in food. The findings on sweet taste preference are consistent with one psychophysical study showing that optimally preferred sucrose concentrations were higher for the elderly than for other age groups, except for the children [50]. As regards fat preferences, it is noted that the ability to accurately assess the fat content of foods is limited in humans, but adults may be more responsive to visual cues indicating the healthiness of food, which could influence food choice [51]. 

### 4.3. Biomarkers

GLP-1 is co-secreted with PYY by L cells in the lower intestine, with concentrations of both hormones increasing in response to a meal and inducing acute satiety [52,53,54]. Deficiencies in GLP-1 and PYY have been reported in obese individuals [55,56,57,58], although not consistently [59,60,61] and not in the present study. We do, however, report higher postprandial concentrations of GLP-1 and PYY in the elderly and in females, which in the long term could partially facilitate weight reduction. Ageing modifies the gastrointestinal tract, causing alterations in gut hormone secretion and feedback mechanisms, which slow gastric emptying [62]. Furthermore, authors have observed slower gastric emptying rates in females compared to males [63,64]. Elevated concentrations of GLP-1 and PYY contribute to delayed gastric emptying and prolonged satiety [56,65]. Therefore, we speculate that slower gastric emptying in the elderly and female groups may have partially accounted for elevated postprandial levels of GLP-1 and PYY. In addition, gastric emptying and satiety hormones have been shown to fluctuate depending on the phase of the menstrual cycle [66,67,68], highlighting the important role of sex hormones in appetite control.

Ghrelin is the only known gastrointestinal hormone to increase food intake [69]. Its concentrations peak prior to meal initiation and are suppressed by nutrient intake [69]. We report elevated fasting ghrelin concentrations in the normal weight compared to overweight participants and in females vs. males, as shown previously [70], however, postprandial patterns of response were similar. Ghrelin concentrations did not differ between adults and elderly, which agrees with previous data [71,72,73,74,75], though findings to the contrary have also been reported [76,77,78,79]. Ghrelin exists as two isoforms: acyl ghrelin, which stimulates energy intake [80,81] and des-acyl ghrelin, which may act independently from acyl ghrelin [82]. Most studies, including the present study, reporting no differences in ghrelin concentrations between younger and older adults [71,72,73,75], have measured total ghrelin (acyl and des-acyl ghrelin) only. However, studies measuring acyl ghrelin observe lower concentrations and an impaired postprandial response in the elderly [77,78,79]. Therefore, the form of ghrelin measured may contribute to discrepancies in the literature and merits further investigation.

Although insulin is predominantly considered a principal regulator of glucose metabolism, it also acts on the arcuate nucleus of the hypothalamus to signal satiety [83]. Studies demonstrating increased satiety with age also report greater postprandial insulin concentrations in older compared to younger adults [73,78,84], in agreement with our findings. Insulin modulates changes in the circulation of leptin [85] and ghrelin [86] and may induce satiety indirectly by amplifying the anorectic actions of leptin and/or suppressing ghrelin secretion [86,87]. In the present study, we did not observe differences in leptin or ghrelin concentrations between adults and the elderly, suggesting that greater insulin secretion in the elderly was not sufficient to cause age-related differences in leptin and ghrelin release.

This is one of the few studies to compare appetite control in different geographical locations. We observed lower ghrelin concentrations in HUA participants compared to those in ABDN. However, differences at baseline were accountable for postprandial differences between each “site”. Interestingly, we also observed elevated fasting leptin concentrations and postprandial insulin concentrations in the HUA cohort compared to the ABDN cohort, which may have partially modulated ghrelin expression. These are novel findings and not likely due to technical issues since processing, storage and analysis were identical, instead, differences in leptin and insulin levels appear to be associated with differences in body composition between the two locations, though body composition did not account for differences in ghrelin concentrations and neither did gender. Future studies may consider the influence of geographical location on variations in appetite control, as other authors have suggested that differences are not related to habitual diet [88].

In the current study, we observed a significant decrease in ghrelin concentrations in response to the caloric load, but not the protein amount. This lack of protein-induced dose-dependent effect is reported by other authors [25,89]. It has been suggest that the postprandial decrease in ghrelin may be mediated through stimulation of gastric inhibitory polypeptide (GIP) and glucagon [25,90,91], possibly linked to gastric emptying, but also the carbohydrate content of the meal [25,91]. We also suggest that interactions between the protein and carbohydrate content is likely to influence ghrelin release, and that dietary carbohydrate may be a more potent stimulator than protein. Interestingly, in the current study, the HP drinks increased GLP-1 and PYY in comparison to the NP drinks. Belza et al. [25] provide a concise commentary on this aspect and suggest that these two hormones, in combination, do affect appetite after a protein-rich meal.

### 4.4. Strengths and Limitations

The present study has several strengths, which include having a controlled diet intervention study conducted as a randomized crossover design in a large cohort taking account of age, body size, gender and geographical location. For some factors (such as drink), this study has the strengths of a crossover design, whereas for others (such as age), there are the unavoidable limitations of studying observable factors. As with any lab-based dietary intervention study, there are limitations, such as limited ecological validity, the amount and type of protein and many phenotypic effects which have not been investigated. The iso-energetic load for the meals was achieved by reducing carbohydrate content, so we cannot rule out the effect of this lower carbohydrate nutrient profile of the high-protein meal to contribute to the study results. We presented unadjusted *p*-values for comparing treatment groups in tests of several variables, to preserve the power of the study, so although there are clear patterns of significant differences, there is a risk that a small proportion of these are type I errors. Therefore, significant *p*-values presented within the present study come with this caveat, which should be considered when interpreting our findings. We recruited people motivated to respond to a diet trial and, consequently, this is not a truly random sample. Furthermore, had we recruited a much larger sample size, this may have allowed the statistical results to be generalized to a larger population or phenotype. Finally, long-term intervention and monitoring across the lifecourse to assess the mechanisms underlying changes in appetite control were out with the scope of this study.

## 5. Conclusions

The primary aim of this study was to describe the acute regulation of appetite across the lifecourse, thus being able to detect differences between four different age groups (children, adolescents, adult and elderly), two different weight categories (normal weight and overweight), the two genders (male and female) and two European sites (Aberdeen, Scotland and Athens, Greece). The present study shows that the elderly reported lower subjective appetite ratings in response to the different drink types and quantities in comparison to the adults. Furthermore, in agreement with Di Francesco et al. [73], postprandial anorexigenic signals prevailed over orexigenic signals in the elderly, which over time could induce an energy deficit and accentuate the anorexia of ageing. In addition to age, differences in appetite hormone concentrations between BMIs, genders and geographical locations were also observed, the latter of which being of particular interest, as location is rarely considered in the context of acute appetite control. Future research may consider expanding upon our findings and examining the role of appetitive neuronal circuits in food–gut–brain interactions across the lifecourse.

## Figures and Tables

**Table 1:** Composition of the high-protein and normal-protein test drinks per 100 mL compared to whole milk.

Product (per 100 mL)		HP Drink	NP Drink	Whole Milk
Total Energy	(kcal)	130	130	63
Protein	(g)	10.0	5.0	3.4
	Energy (%)	30.7	15.3	21.9
	Casein (g)	8.0	4.0	2.7
	Whey (g)	2.0	1.0	0.7
Fat	(g)	3.5	3.5	3.6
	Energy (%)	24.2	24.2	50.6
Carbohydrate	(g)	14.7	19.7	4.6
	Energy (%)	45.1	60.5	27.9
	Lactose (g)	<0.06	<0.06	4.6

HP: high-protein test drink, NP: normal-protein test drink.

**Table 2:** Participant characteristics by age group and site ^1^.

	Children			Adolescents			Adults			Elderly		
	ABDN (*n* = 39)	HUA (*n* = 64)	All (*n* = 103)	ABDN (*n* = 45)	HUA (*n* = 64)	All (*n* = 109)	ABDN (*n* = 46)	HUA (*n* = 51)	All (*n* = 97)	ABDN (*n* = 36)	HUA (*n* = 46)	All (*n* = 82)
Age (years)	8.72 ± 0.69	9.20 ± 0.65	9.02 ± 0.70	15.4 ± 1.28	14.5 ± 1.33	14.9 ± 1.37	29.9 ± 7.34	32.8 ± 6.71	31.4 ± 7.13	68.0 ± 3.82	68.5 ± 3.88	68.3 ± 3.84
Height (m)	1.37 ± 0.08	1.40 ± 0.06	1.39 ± 0.07	1.65 ± 0.09	1.67 ± 0.09	1.66 ± 0.09	1.71 ± 0.09	1.71 ± 0.10	1.71 ± 0.09	1.64 ± 0.08	1.62 ± 0.08	1.62 ± 0.08
Weight (kg)	31.6 ± 6.94	38.3 ± 8.39	35.7 ± 8.49	60.3 ± 12.3	65.7 ± 10.4	63.4 ± 11.5	71.0 ± 14.2	76.1 ± 14.4	73.7 ± 14.5	68.6 ± 11.9	75.7 ± 14.4	72.6 ± 13.7
BMI (kg/m^2^)	16.7 ± 2.41	19.5 ± 3.33	18.4 ± 3.31	22.2 ± 4.23	23.5 ± 2.82	23.0 ± 3.51	24.3 ± 4.07	25.9 ± 4.33	25.1 ± 4.27	25.5 ± 3.58	28.9 ± 4.71	27.4 ± 4.55
BMR ^2^ (MJ)	4.93 ± 0.62	5.52 ± 0.82	5.30 ± 0.8	6.70 ± 1.02	7.14 ± 1.04	6.96 ± 1.05	6.67 ± 1.04	7.18 ± 1.17	6.94 ± 1.13	5.73 ± 0.74	6.54 ± 1.00	6.19 ± 0.98
Body fat ^3^ (%)	22.8 ± 5.70	25.4 ± 5.93	24.4 ± 5.95	24.8 ± 8.47	24.8 ± 7.54	24.8 ± 7.90	23.6 ± 10.1	26.2 ± 9.95	24.9 ± 10.1	32.4 ± 6.78	35.4 ± 6.78	34.2 ± 6.90
Waist Circumference ^4^ (cm)	64.5 ± 8.56	71.8 ± 12.4	69.6 ± 11.8	81.6 ± 10.9	84.9 ± 9.75	83.7 ± 10.3	91.4 ± 13.0	96.9 ± 12.4	94.4 ± 12.9	100 ± 14.0	109 ± 12.8	106 ± 14.0
Visceral Fat ^4^ (%)	3.67 ± 2.23	4.95 ± 3.25	4.58 ± 3.03	5.43 ± 3.67	6.05 ± 3.28	5.81 ± 3.43	7.72 ± 4.92	9.64 ± 4.64	8.76 ± 4.84	10.4 ± 4.25	15.1 ± 6.76	13.2 ± 6.29

^1^ Values are means ± SD; ^2^ calculated by Schofield equation; ^3^ measured by whole body BIA; ^4^ measured by abdominal VISCAN bio-impedance; ABDN: Scotland, BIA: bioelectrical impedance analysis, BMR: basal metabolic rate, HUA: Greece.

**Table 3:** Test drink energy and protein consumption ^1^.

		NPWL	NPMT	HPWL	HPMT	SED_type_	*p*_type_^2^
Energy (kcal)	Children (*n* = 102)	306	425	304	425	10	<0.001
	Adolescents (*n* = 108)	406	565	402	557		
	Adults (*n* = 97)	399	553	395	548		
	Elderly (*n* = 82)	348	483	343	478		
Protein (g)	Children (*n* = 102)	11.4	15.9	23.3	32.6	0.6	<0.001
	Adolescents (*n* = 108)	15.2	21.1	30.8	42.7		
	Adults (*n* = 97)	14.9	20.6	30.3	42.0		
	Elderly (*n* = 82)	13.0	18.0	26.3	36.6		

^1^ Corrected for mass consumed. Mean data are presented for drink type (NPWL, NPMT, HPWL, HPMT); ^2^ determined by ANOVA, differences are statistically significant when *p* < 0.05; HPMT: high-protein maintenance, HPWL: high-protein weight loss, NPMT: normal-protein maintenance, NPWL: normal-protein weight loss.

**Table 4:** Ad libitum EI (kcal) ^1^.

Group		NPWL	NPMT	HPWL	HPMT	SED_type_	*p*_type_^2^	NP	HP	SED_composition_	*p*_composition_^2^	WL	MT	SED_quantity_	*p*_quantity_^2^
Age	Children (*n* = 103)	718	641	679	646	29	0.483	679	663	21	0.941	699	644	21	0.074
	Adolescents (*n* = 109)	950	876	940	852			914	895			945	864		
	Adults (*n* = 97)	672	603	641	627			639	634			658	615		
	Elderly (*n* = 82)	526	522	478	500			524	490			502	510		
BMI	Normal Weight (*n* = 221)	701	644	656	636	21	0.928	672	646	15	0.700	677	639	15	0.858
	Overweight (*n* = 170)	732	677	715	675			706	696			725	677		
Gender	Males (*n* = 171)	825	751	773	744	21	0.166	789	758	15	0.220	799	749	15	0.123
	Females (*n* = 220)	608	569	598	567			589	581			603	567		
Site	HUA (*n* = 225)	806	766	797	756	21	0.245	787	778	15	0.272	801	761	15	0.897
	ABDN (*n* = 166)	627	555	572	555			591	565			600	555		

^1^*n* = 391. Mean data are presented for drink type (NPWL, NPMT, HPWL, HPMT), drink composition (NP, HP) and drink quantity (WL, MT); ^2^ determined by ANOVA (with age, BMI, gender and site as fixed factors), differences are statistically significant when *p* < 0.05; ABDN: Scotland, EI: energy intake, HP: high protein, HPMT: high-protein maintenance, HPWL: high-protein weight loss, HUA: Greece, MT: weight maintenance, NP: normal protein, NPMT: normal-protein maintenance, NPWL: normal-protein weight loss, WL: weight loss.

**Table 5:** Total caloric intake (kcal) ^1^.

Group		NPWL	NPMT	HPWL	HPMT	SED_type_	*p*_type_^2^	NP	HP	SED_composition_	*p*_composition_^2^	WL	MT	SED_quantity_	*p*_quantity_^2^
Age	Children (*n* = 103)	1022	1075	991	1077	50	0.562	1049	1034	46	0.793	1007	1076	46	0.143
	Adolescents (*n* = 109)	1372	1461	1352	1416			1416	1384			1362	1438		
	Adults (*n* = 97)	1078	1168	1048	1186			1123	1117			1063	1177		
	Elderly (*n* = 82)	885	1022	835	993			953	914			860	1008		
BMI	Normal Weight (*n* = 221)	1235	1325	1,85	1314	31	0.625	1280	1249	29	0.499	1210	1319	29	0.940
	Overweight (*n* = 170)	943	1038	928	1023			991	975			936	1030		
Gender	Males (*n* = 171)	1044	1126	996	1112	31	0.775	1085	1054	29	0.751	1020	1119	29	0.455
	Females (*n* = 220)	1134	1237	1117	1225			1185	1171			1125	1231		
Site	HUA (*n* = 225)	995	1075	942	1063	30	0.287	1035	1002	28	0.239	968	1069	28	0.862
	ABDN (*n* = 166)	1183	1288	1171	1274			1236	1222			1177	1281		

^1^*n* = 391. Mean data are presented for drink type (NPWL, NPMT, HPWL, HPMT), drink composition (NP, HP) and drink quantity (WL, MT); ^2^ determined by ANOVA (with age, BMI, gender and site as fixed factors), differences are statistically significant when *p* < 0.05; ABDN: Scotland, EI: energy intake, HP: high protein, HPMT: high-protein maintenance, HPWL: high-protein weight loss, HUA: Greece, MT: weight maintenance, NP: normal protein, NPMT: normal-protein maintenance, NPWL: normal-protein weight loss, WL: weight loss.

**Table 6:** Children ^1^ and adolescent ^2^ motivation to eat at baseline and in response to test drink type, composition and quantity.

		Time (mins)	NPWL	NPMT	HPWL	HPMT	SED_type_	Type.Time Interaction	NP	HP	SED_composition_	Composition.Time Interaction	WL	MT	SED_quantity_	Quantity.Time Interaction
								*p* ^3^				*p* ^3^				*p* ^3^
Fullness	Children	0	2.25	2.02	1.77	2.19	0.21	0.252	2.13	1.98	0.16	0.220	2.01	2.10	0.16	0.554
		30	3.73	3.86	3.37	3.71			3.79	3.54			3.55	3.78		
		60	3.06	3.14	3.02	2.83			3.10	2.92			3.04	2.98		
		90	2.60	2.75	2.52	2.51			2.68	2.51			2.56	2.63		
		120	2.13	2.28	2.33	2.19			2.21	2.26			2.23	2.23		
	Adolescents	0	2.69	2.90	2.95	2.95	0.18		2.80	2.95	0.14		2.82	2.92	0.14	
		30	4.49	4.85	5.06	5.13			4.67	5.10			4.78	4.99		
		60	4.15	4.44	4.52	4.59			4.29	4.56			4.33	4.52		
		90	3.65	3.94	3.91	4.06			3.80	3.98			3.78	4.00		
		120	3.28	3.53	3.59	3.59			3.40	3.59			3.43	3.56		
PFC	Children	0	6.61	6.89	7.05	7.16	0.19	0.332	6.75	7.10	0.16	0.209	6.83	7.02	0.16	0.653
		30	6.00	6.00	6.22	5.90			6.00	6.06			6.11	5.95		
		60	6.70	6.55	6.54	6.96			6.62	6.75			6.62	6.76		
		90	7.14	7.14	7.05	7.26			7.14	7.16			7.10	7.20		
		120	7.62	7.65	7.55	7.60			7.64	7.57			7.58	7.63		
	Adolescents	0	6.19	6.06	6.05	5.90	0.21		6.13	5.98	0.14		6.12	5.98	0.14	
		30	5.08	4.63	4.58	4.31			4.85	4.44			4.83	4.47		
		60	5.47	5.05	5.08	4.96			5.26	5.02			5.27	5.01		
		90	5.85	5.66	5.61	5.61			5.76	5.61			5.73	5.64		
		120	6.42	6.08	6.07	6.10			6.25	6.09			6.25	6.09		

^1^*n* = 103 for children; ^2^ *n* = 109 for adolescents; ^3^ determined by ANOVA between age groups, differences are statistically significant when *p* < 0.05. Mean data are presented for drink type (NPWL, NPMT, HPWL, HPMT), drink composition (NP, HP) and drink quantity (WL, MT); HP: high protein, HPMT: high-protein maintenance, HPWL: high-protein weight loss, MT: weight maintenance, NP: normal protein, NPMT: normal-protein maintenance, NPWL: normal-protein weight loss, PFC: prospective food consumption, WL: weight loss.

**Table 7:** Adult^1^ and elderly^2^ composite appetite scores at baseline and in response to test drink type, composition and quantity.

		Time (mins)	NPWL	NPMT	HPWL	HPMT	SED_type_	Type.Time Interaction	NP	HP	SED_composition_	Composition.Time Interaction	WL	MT	SED_quantity_	Quantity.Time Interaction
								*p* ^3^				*p* ^3^				*p* ^3^
Appetite Score (mm)	Adults	0	63.2	65.6	61.5	62.8	2.1	0.004	64.4	62.1	1.6	0.624	62.3	64.2	1.6	< 0.001
		30	42.6	35.5	41.2	34.8			39.0	38.0			41.9	35.1		
		60	46.7	40.3	44.6	37.9			43.5	41.3			45.7	39.1		
		90	52.0	44.8	49.6	42.4			48.4	46.0			50.8	43.6		
		120	55.9	50.9	54.6	48.1			53.4	51.4			55.3	49.5		
	Elderly	0	44.5	43.7	45.4	43.9	2.7		44.1	44.6	2.1		44.9	43.8	2.1	
		30	31.4	28.6	29.7	31.3			30.0	30.5			30.5	30.0		
		60	35.2	33.7	34.7	33.0			34.4	33.9			35.0	33.3		
		90	40.9	37.5	39.1	36.5			39.2	37.8			40.0	37.0		
		120	44.3	41.9	43.2	38.3			43.1	40.8			43.7	40.1		

^1^*n* = 97 for adults; ^2^ *n* = 82 for elderly; ^3^ determined by ANOVA between age groups, differences are statistically significant when *p* < 0.05. Mean data are presented for drink type (NPWL, NPMT, HPWL, HPMT), drink composition (NP, HP) and drink quantity (WL, MT); HP: high protein, HPMT: high-protein maintenance, HPWL: high-protein weight loss, MT: weight maintenance, NP: normal protein, NPMT: normal-protein maintenance, NPWL: normal-protein weight loss, VAS: visual analog scale, WL: weight loss.

**Table 8:** Combined adult and elderly^1^ appetite hormone concentrations at baseline and in response to test drink type, composition and quantity.

Drink	Time (min)	GLP-1 (pg/mL)	SED	Drink.Time Interaction, *p* ^2^	PYY (pg/mL)	SED	Drink.Time Interaction, *p* ^2^	Ghrelin (pg/mL)	SED	Drink.Time Interaction, *p* ^2^
NPWL	0	12.7	1.3	<0.001	46.1	1.9	<0.001	773	12	<0.005
	30	33.1			68.3			651		
	60	26.6			69.6			596		
	120	21.8			65.2			604		
NPMT	0	11.9			43.5			762		
	30	37.1			71.3			659		
	60	31.3			74.3			584		
	120	28.7			73.4			574		
HPWL	0	12.1			44.9			767		
	30	31.3			63.2			661		
	60	27.6			63.4			603		
	120	26.3			64.2			622		
HPMT	0	12.5			45.2			763		
	30	33.9			66.4			653		
	60	31.1			67.3			578		
	120	28.7			66.5			572		
NP	0	12.3	1.1	<0.001	44.8	1.5	<0.001	767	9	0.710
	30	35.1			69.8			655		
	60	29.0			72.0			590		
	120	25.2			69.3			589		
HP	0	12.3			45.1			765		
	30	32.6			64.8			657		
	60	29.4			65.4			591		
	120	27.5			65.3			597		
WL	0	12.4	1.1	<0.001	45.5	1.5	<0.001	770	9	< 0.001
	30	32.2			65.7			656		
	60	27.1			66.5			599		
	120	24.1			64.7			613		
MT	0	12.2			44.4			763		
	30	35.5			68.8			656		
	60	31.2			70.8			581		
	120	28.7			69.9			573		

^1^*n* = 172; seven ABDN participants (four normal weight adults, four normal weight elderly) did not complete this measurement; ^2^ determined by ANOVA, differences are statistically significant when *p* < 0.05. Mean data are presented for drink type (NPWL, NPMT, HPWL, HPMT), drink composition (NP, HP) and drink quantity (WL, MT); GLP-1: glucagon-like peptide 1, HP: high protein, HPMT: high-protein maintenance, HPWL: high-protein weight loss, MT: weight maintenance, NP: normal protein, NPMT: normal-protein maintenance, NPWL: normal-protein weight loss, PYY: pancreatic peptide YY, WL: weight loss.

## Supplementary Materials

The following are available online at https://www.mdpi.com/2072-6643/12/12/3710/s1, Table S1: Schofield equations for estimating BMR, Table S2: Nutritional values of the foods and drinks served at the ad libitum buffet-style test meal, Table S3: Number of participants completed within each group at both sites, Table S4: Buffet-style test meal ad libitum energy and macronutrient intake (*n* = 391), Table S5: Effect of visit number on ad libitum EI, Table S6: Mean food reward (liking and wanting) results, Table S7: Combined adult and elderly glucose homeostasis, by group, at baseline and post-test drink consumption, Table S8: Combined adult and elderly glucose homeostasis at baseline and in response to test drink type, composition and quantity, Figure S1: CONSORT diagram summarizing participant flow. The number of participants from both study sites who were recruited, enrolled, allocated to intervention, discontinued, included in the analyses and completed are presented. ABDN: Scotland, HUA: Greece.
