# Supplementary material for: Appetite Control across the Lifecourse: The Acute Impact of Breakfast Drink Quantity and Protein Content. The Full4Health Project
Source: Nutrients. 2020 Nov 30;12(12):3710. doi: 10.3390/nu12123710 (PMC7759987; doi:10.3390/nu12123710)
Supplement: Supplementary file 1 [file nutrients-12-03710-s001.pdf]

Supplementary files

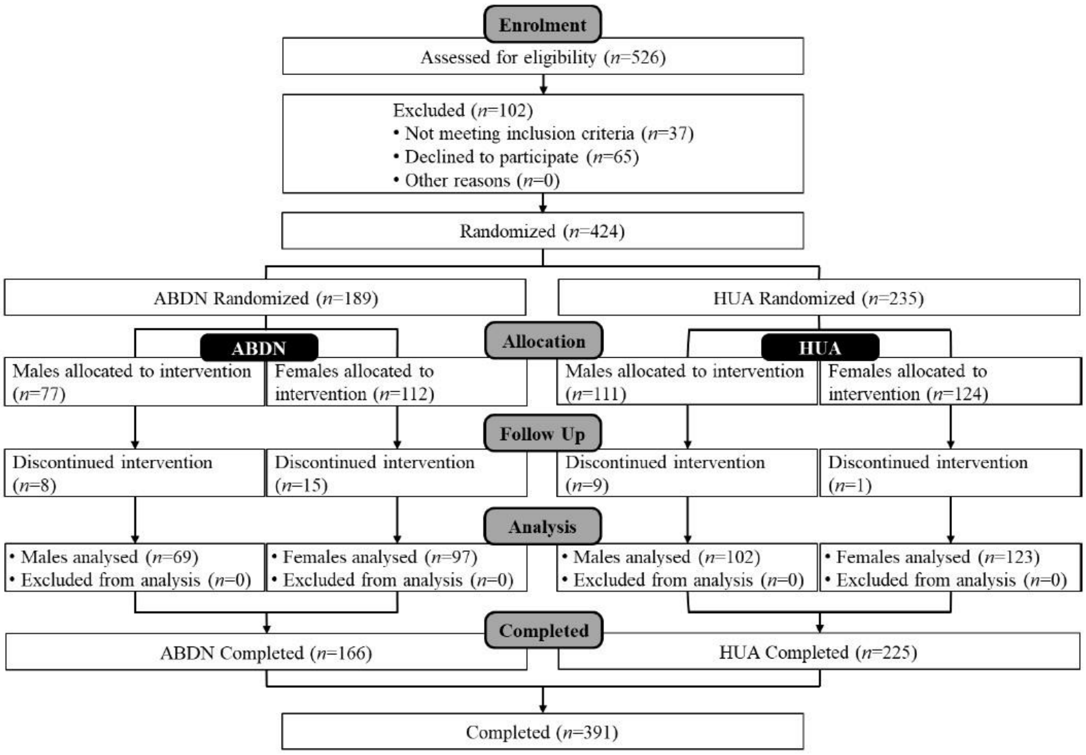

Figure S1. CONSORT diagram summarizing participant flow. The number of participants from both study sites who were recruited, enrolled, allocated to intervention, discontinued, included in the analyses and completed are presented. (ABDN) Scotland, (HUA) Greece.

Table S1. Schofield equations for estimating BMR.

Gender	Age range (years)	BMR equation (MJ)
**Males**	8-10	BMR = 0.095W + 2.110
	10-17	BMR = 0.074W + 2.754
	18-29	BMR = 0.063W + 2.896
	30-59	BMR = 0.048W + 3.653
	60-74	BMR = 0.0499W + 2.930
**Females**	8-10	BMR = 0.085W + 2.033
	10-17	BMR = 0.056W + 2.898
	18-29	BMR = 0.062W + 2.036
	30-59	BMR = 0.034W + 3.538
	60-74	BMR = 0.0386W + 2.875

(BMR), basal metabolic rate, (W) body weight.

Table S2. Nutritional values of the foods and drinks served at the *ad libitum* buffet-style test meal.

	Food or drink products	Portion size	Energy (kcal)	Protein (g)	CHO (g)	Sugars (g)	NMES (g)	Fat (g)	Sat Fat (g)	Salt (g)
High-fat savory	Full fat cheese	60g	188.1	13.5	0.1	0.0	0.0	15.0	0.0	0.0
	Full-fat cheese spread sandwich, white bread	70g	164	4.8	19.7	2.2	1.2	7.7	4.9	0.6
	Crackers	18g	89.5	1.3	10.2	1.3	0.0	5.1	3.8	0.5
	Ready salted potato crisps	65g	334.8	4.2	31.9	0.3	0.0	22.1	1.7	1.0
Low-fat savory	Carrot sticks	80g	27.9	0.5	6.3	5.9	0.0	0.2	0.1	0.1
	Cucumber sticks	80g	7.9	0.6	1.2	1.1	0.0	0.1	0.0	0.0
	Breadsticks	25g	99.2	2.9	18.2	0.9	0.0	2.0	1.7	0.5
	Rice Cakes	21g	85.0	1.8	18.9	0.3	0.0	0.6	0.3	0.0
Low-fat sweet	Fat-free peach yogurt	125g/200g[1]	70.0	6.0	11.6	11.4	0.0	0.1	0.0	0.2
	Strawberry yogurt	125g/200g[1]	113.0	4.4	15.6	15.3	0.0	4.0	2.5	0.2
	Apples	300g/2 apples[1]	155.4	0.9	39.0	39.0	0.0	0.3	0.0	0.0
	Raisins	80g	221.6	1.7	55.4	55.4	55.4	0.3	0.0	0.1
	Cereal Bar	23g	85.7	1.8	17.7	8.7	0.0	1.2	0.8	0.1
High-fat sweet	Toblerone chocolate bar	100g	510.9	5.6	60.0	60.0	0.0	29.2	17.2	0.1
	Kit Kat chocolate biscuit	96g	480.2	7.9	58.1	44.9	44.9	25.5	13.2	0.3
	Mars bar	54g	239.5	2.9	35.9	35.5	35.5	10.2	5.4	0.2
	Chocolate Brownie/ Chocolate Cookies[1]	80g	335.8	4.8	44.9	35.4	0.0	16.3	9.8	0.4
Beverages	Semi-skimmed milk	300g	140.2	9.9	15.0	15.0	0.0	4.8	3.0	0.4
	Orange juice	400mL/1000mL[1]	146.4	2.0	35.2	35.2	35.2	0.4	0.0	0.1
	Apple juice	400mL/1000mL[1]	156.7	0.4	39.6	39.6	39.6	0.4	0.0	0.0
	Water, distilled	500mL	0.0	0.0	0.0	0.0	0.0	0.0	0.0	0.0
	Cola	330mL	135.0	0.0	34.7	34.7	34.7	0.0	0.0	0.1
	Diet Coke	330mL	0.0	0.0	0.0	0.0	0.0	0.0	0.0	0.0
	**Total**	-	3783.7	77.9	569.2	442.0	246.5	145.5	64.4	4.8

[1] Alternative HUA weights or food products; (CHO) carbohydrates, (HUA) Greece, (NMES) non-milk extrinsic sugars, (Sat Fat) saturated fat.

Table S3. Number of participants completed within each group at both sites.

Group	ABDN	HUA
8-10 years, normal-weight females	10	16
8-10 years, overweight females	7	16
8-10 years, normal-weight males	17	16
8-10 years, overweight males	5	16
13-17 years, normal-weight females	17	16
13-17 years, overweight females	9	16
13-17 years, normal-weight males	16	16
13-17 years, overweight males	3	16
25-45 years, normal-weight females	16	16
25-45 years, overweight females	10	12
25-45 years, normal-weight males	13	15
25-45 years, overweight males	7	8
65-75 years, normal-weight females	17	15
65-75 years, overweight females	11	16
65-75 years, normal-weight males	3	2
65-75 years, overweight males	5	13
**Total**	**166**	**225**

(ABDN) Scotland, (HUA) Greece.

Table S4. Buffet-style test meal *ad libitum* energy and macronutrient intake for all participants combined[1].

ALL		NPWL	NPMT	HPWL	HPMT	SED_{type}	p_{type2}	NP	HP	$SED_{composition}$	$p_{composition^1}$	WL	MT	$SED_{quantity}$	$p_{quantity2}$
**Energy**	kcal	717	661	685	656	15	< 0.001	689	670	11	0.168	701	658	11	< 0.001
**Fat**	g	27.99	25.47	26.70	25.63	0.72	< 0.001	26.73	26.17	0.51	0.390	27.35	25.55	0.51	< 0.001
	kcal	248	225	236	227	6		237	232	5		242	226	5	
	%	33.20	32.70	32.63	33.25	0.56	0.326	32.95	32.94	0.40	0.961	32.92	32.98	0.40	0.202
**Protein**	g	17.90	16.70	17.38	16.47	0.39	< 0.001	17.30	16.92	0.27	0.251	17.64	16.58	0.27	< 0.001
	kcal	73	68	67	71	2		70	69	1		72	67	1	
	%	10.80	10.78	10.91	11.00	2.56	0.929	10.79	10.96	0.18	0.522	10.85	10.89	0.18	0.917
**CHO**	g	101.37	93.96	96.81	92.57	2.12	< 0.001	97.67	94.69	1.50	0.112	99.10	93.26	1.50	< 0.001
	kcal	388	360	371	354	8		374	362	6		379	357	6	
	%	54.84	55.27	55.23	54.51	0.59	0.416	55.06	54.87	0.42	0.783	55.04	54.89	0.42	0.288
**Fiber**	g	4.12	3.91	3.99	3.82	0.10	0.002	4.01	3.91	0.07	0.295	4.05	3.86	0.07	< 0.001
	kcal	8	7	8	7	0		8	7	0		8	7	0	
	%	1.16	1.25	1.22	1.24	0.05	0.332	1.20	1.23	0.03	0.535	1.19	1.24	0.03	0.169

[1] $n = 391$; [2] Determined by ANOVA, differences are statistically significant when $p < 0.05$; Mean data are presented for drink type (NPWL, NPMT, HPWL, HPMT), drink composition (NP, HP) and drink quantity (WL vs MT); (CHO) Carbohydrates, (HP) high protein, (HPMT) high-protein maintenance, (HPWL) high-protein weight loss, (MT) weight maintenance, (NP) normal protein, (NPMT) normal-protein maintenance, (NPWL) normal-protein weight loss, (WL) weight loss.

Table S5. Effect of visit number on *ad libitum* EI[1].

	Children ($n = 103$)	Adolescents ($n = 109$)	Adults ($n = 97$)	Elderly ($n = 82$)	SED	$p_{Age.Visit^1}$	All	SED	p_{Visit}
**Visit 1**	706	886	548	430	53.4	< 0.001	659[a]	13.1	0.001
**Visit 2**	663	957	623	492			699[b]		
**Visit 3**	684	962	641	478			707[b]		
**Visit 4**	661	886	640	518			688[b]		

[1]Mean data are presented for each age group and all age groups combined ($n = 391$); [1] Determined by ANOVA (with age as fixed factor), differences are statistically significant when $p < 0.05$; [a, b] Mean values within a row with unlike superscript letters are significantly different.

Table S6. Mean food reward (liking and wanting) results[1].

	Group	Liking						Wanting					
		Fat[2]	SEM	p[3]	Sweet[4]	SEM	p[3]	Fat[2,5]	SEM	p[3]	Sweet[4,6]	SEM	p[3]
SITE	HUA (n = 225)	-5.55	1.50	< 0.001	3.38	1.35	0.019	-14.38	2.48	< 0.001	3.97	3.18	0.019
	ABDN (n = 164)	1.81	1.27		8.32	1.60		5.19	2.93		14.70	3.26	
GENDER	Males (n = 169)	-1.33	1.60	0.585	5.91	1.70	0.953	-3.84	3.12	0.693	11.69	3.82	0.301
	Females (n= 220)	-2.41	1.14		5.79	1.21		-5.36	2.23		6.97	2.48	
BMI	Normal Weight (n = 220)	-3.36	1.36	0.131	7.93	1.44	0.047	-7.21	2.65	0.174	13.65	3.34	0.059
	Overweight (n = 169)	-0.38	1.42		3.77	1.51		-1.98	2.78		5.01	3.10	
AGE	Children (n = 101)	-1.51	1.78	0.001	7.91	1.89	0.093	3.98[a]	3.49	< 0.001	16.70[a]	3.88	< 0.001
	Adolescents (n = 109)	4.22[a]	1.85		6.42	1.96		6.89[a]	3.60		12.34[a]	4.01	
	Adults (n = 97)	-3.11[b]	1.76		1.71	1.87		-12.07[b]	3.43		-5.99[b]	3.82	
	Elderly (n = 82)	-7.08[b]	2.41		7.35	2.56		-17.17[b]	4.70		14.27[a]	6.10	

Values are mean ± SEM. [1]n = 389; [2] Positive scores indicate greater liking/wanting for high-fat relative to low-fat foods, whereas negative scores indicate greater liking/wanting for low-fat relative to high-fat foods; [3] Determined by using univariate ANOVA (with site, gender, BMI and age as fixed factors), differences are statistically significant when $p < 0.05$. Unalike letters indicate significant difference in post hoc analyses with Bonferroni adjustment; [4] Positive scores indicate greater liking/wanting for sweet relative to savory foods, whereas negative scores indicate greater liking/wanting for savory relative to sweet foods; [5] Two extreme outliers were removed from the analyses of this parameter: both HUA, children and overweight, 1 female and 1 male; [6] Two extreme outliers were removed from the analyses of this parameter: both HUA, male, 1 elderly lean and 1 child with overweight; (ABDN) Scotland, (HUA) Greece.

Table S7. Combined adult and elderly glucose homeostasis, by group, at baseline and post-test drink consumption[1].

	Group	Time (mins)	Glucose (mmol/L)	SED	p^2	Insulin (pmol/L)	SED	p^2	HOMA-IR	SED	p^2	IGI	SED	p^2	IGR	SED	p^2
**AGE**	Adults (*n* = 93)	0	5.48	0.21	< 0.001	44.0	34.0	< 0.001	0.002	0.001	< 0.001	0.061	0.082	0.450	0.0008	0.0005	< 0.001
		30	6.58			535			0.023			0.588			0.0100		
		60	5.18			421			0.015			0.884			0.0106		
		120	5.07			223			0.008			0.391			0.0051		
	Elderly (*n* = 79)	0	5.77			56.6			0.002			0.055			0.0016		
		30	7.07			536			0.024			0.457			0.0123		
		60	6.32			596			0.026			0.848			0.0135		
		120	5.81			353			0.015			0.447			0.0083		
**BMI**	Normal Weight (*n* = 90)	0	5.39	0.22	< 0.001	32.3	34.3	< 0.001	0.001	0.001	< 0.001	0.040	0.083	0.903	0.0011	0.0005	0.006
		30	6.56			475			0.020			0.488			0.0115		
		60	5.18			409			0.014			0.867			0.0112		
		120	4.87			195			0.007			0.382			0.0059		
	Overweight (*n* = 82)	0	5.86			69.1			0.003			0.079			0.0013		
		30	7.07			602			0.027			0.571			0.0107		
		60	6.28			602			0.025			0.868			0.0129		
		120	6.00			379			0.016			0.454			0.0075		
**GENDER**	Males (*n*= 64)	0	5.57	0.22	0.036	51.9	35.3	0.097	0.002	0.002	0.212	0.060	0.085	0.346	0.0012	0.0005	0.005
		30	6.61			519			0.024			0.421			0.0100		
		60	5.53			546			0.022			0.798			0.0114		
		120	5.28			324			0.014			0.428			0.0071		
	Females (*n* = 108)	0	5.69			48.6			0.002			0.057			0.0012		
		30	7.13			546			0.022			0.591			0.0117		
		60	6.00			492			0.018			0.909			0.0123		
		120	5.63			258			0.010			0.409			0.0063		
**SITE**	HUA (*n* = 97)	0	5.60	0.21	0.008	51.3	34.2	0.005	0.002	0.001	0.017	0.055	0.083	0.084	0.0012	0.0005	0.005
		30	6.83			563			0.024			0.538			0.0116		
		60	5.82			560			0.022			0.974			0.0131		
		120	5.31			303			0.012			0.503			0.0071		
	ABDN (*n* = 75)	0	5.63			47.9			0.002			0.062			0.0012		
		30	6.76			500			0.021			0.514			0.0104		
		60	5.56			425			0.016			0.731			0.0105		

120	5.54	255	0.010	0.305	0.0060

[1] Data are presented as mean concentrations, $n = 172$; 7 ABDN participants (4 normal weight adults, 3 normal weight elderly) did not complete this measurement; [2] Determined by ANOVA, Group.Time interactions (p) are statistically significant when $p < 0.05$; (ABDN) Scotland, (HOMA-IR) homeostatic model assessment for insulin resistance, (HUA) Greece, (IGI) insulinogenic index, (IGR) Insulin-Glucose Ratio.

Table S8. Combined adult and elderly[1] glucose homeostasis at baseline and in response to test drink type, composition and quantity.

Drink		Time (mins)	Glucose (mmol/L)	SED	*p*[2]	Insulin (pmol/L)	SED	*p*[2]	HOMA-IR	SED	*p*[2]	IGI	SED	*p*[2]	IGR	SED	*p*[2]
**Type**	NPWL	0	5.64			50.3			0.002			0.054			0.0012		
		30	7.20			538			0.025			0.438			0.0104		
		60	6.06			488			0.020			0.533			0.0110		
		120	5.24			230			0.009			0.341			0.0054		
	NPMT	0	5.61			49.1			0.002			0.063			0.0012		
		30	7.09			548			0.025			0.473			0.0109		
		60	6.10			550			0.023			0.922			0.0124		
		120	5.90	0.10	< 0.001	401	22.7	< 0.001	0.017	0.001	< 0.001	0.478	0.114	< 0.001	0.0090	0.0005	< 0.001
	HPWL	0	5.61			48.8			0.002			0.061			0.0012		
		30	6.41			492			0.020			0.568			0.0107		
		60	5.30			442			0.017			0.734			0.0111		
		120	5.03			175			0.007			0.330			0.0043		
	HPMT	0	5.60			51.1			0.002			0.055			0.0012		
		30	6.51			565			0.023			0.632			0.0123		
		60	5.36			524			0.019			1.282			0.0134		
		120	5.46			324			0.013			0.515			0.0077		
**Composition**	NP	0	5.62			49.7			0.002			0.059			0.0012		
		30	7.15			543			0.025			0.455			0.0107		
		60	6.08			519			0.021			0.727			0.0117		
		120	5.57	0.08	< 0.001	316	20.0	< 0.001	0.013	0.001	< 0.001	0.410	0.082	0.040	0.0072	0.0004	< 0.001
	HP	0	5.61			50.0			0.002			0.058			0.0012		
		30	6.46			528			0.022			0.600			0.0115		
		60	5.33			483			0.018			1.008			0.0123		
		120	5.25			250			0.010			0.423			0.0060		
**Quantity**	WL	0	5.63			49.5			0.002			0.058			0.0012		
		30	6.81			515			0.022			0.503			0.0106		
		60	5.68	0.08	< 0.001	465	20.0	< 0.001	0.018	0.001	< 0.001	0.634	0.082	< 0.001	0.0111	0.0004	< 0.001
		120	5.14			203			0.008			0.336			0.0049		
	MT	0	5.60			50.1			0.002			0.059			0.0012		
		30	6.80			557			0.024			0.552			0.0116		

60	5.73	537	0.021	1.101	0.0129
120	5.68	363	0.015	0.497	0.0084

[1] Data are presented as mean concentrations, n = 172; 7 ABDN participants (4 normal weight adults, 3 normal weight elderly) did not complete this measurement; [2] Determined by ANOVA, Drink.Time interactions (p) are statistically significant when $p < 0.05$; (ABDN), Scotland, (HOMA-IR) homeostatic model assessment for insulin resistance, (HP) high-protein, (HPMT) high-protein maintenance, (HPWL) high-protein weight loss, (IGI) Insulinogenic index, (IGR) Insulin-Glucose Ratio, (MT) weight maintenance, (NP) normal-protein, (NPMT) normal-protein maintenance, (NPWL) normal-protein weight loss, (WL) weight loss.
